# 2‐line Ferrihydrite Enhance Microbial Synthesis of Plant Biostimulants in Composted Biosolid by Regulating Phyla *Pseudomonadota* and *Actinomycetota*

**DOI:** 10.1002/advs.202506502

**Published:** 2025-12-01

**Authors:** Yu Zhang, Ziwei Zhang, Zexu Chen, Boyuan Yang, Siying Cai, Jun Chen, Jianhua Guo, Weijun Zhang

**Affiliations:** ^1^ School of Environmental Studies China University of Geosciences Wuhan Hubei 430074 China; ^2^ National Engineering Research Center for Industrial Wastewater Detoxication and Resource Recovery Research Center for Eco‐Environmental Sciences Chinese Academy of Sciences Beijing 100085 China; ^3^ Australia Centre for Water and Environmental Biotechnology (ACWEB, formerly AWMC) The University of Queensland St Lucia 4067 Australia

**Keywords:** 2‐line ferrihydrite, aerobic composting, disordered birnessite, plant biostimulants, waste activated sludge

## Abstract

The discovery of plant biostimulants (PBs) in sewage sludge offers a promising avenue for biosolids valorization. Here, the study investigates how two mineral additives, including 2‐line ferrihydrite (a disordered iron oxide) and disordered birnessite (a manganese oxide), modulate microbial activity and molecular pathways to enhance PB production during aerobic sludge composting. Application of 2‐line ferrihydrite significantly promotes the synthesis of growth‐promoting PBs, including arginine, valine, decanoic acid, and indoleacetic acid (IAA), while disordered birnessite primarily enhances resistance‐related PBs, such as decanoic acid, L‐pyroglutamate, and trans‐aconitic acid. In pot trials, composted biosolids amended with 2‐line ferrihydrite significantly improve plant biomass and leaf area compared to mineral‐free and birnessite treatments. Metagenomic profiling reveals that PB biosynthesis is dominated by members of the phyla Pseudomonadota and Actinomycetota, with temporal niche partitioning across the thermophilic and maturation stages. 2‐line ferrihydrite enhances the abundance of critical biosynthetic genes (e.g., *trpA/C/D/E/F*), particularly within taxa such as *Xanthomonadaceae*, *Sphingomonadaceae, and Streptosporangiaceae*. Additionally, genes involved in IAA and indole biosysnthesis (*ALDH*, *DDC*, and *tnaA*) are enriched, supporting enhanced tryptophan‐to‐IAA conversion. This study provides a mechanistic link between iron oxide‐mediated microbial modulation and PB production in composted biosolids, offering a sustainable approach for upgrading waste into high‐value agricultural inputs.

## Graphical abstract

This study explores how iron and manganese oxides transform sewage sludge into plant biostimulants during composting. Non‐targeted identification reveals the main species of plant biostimulants. Metagenomic analysis reveals that 2‐line ferrihydrite specifically enriches microbial genes for biosynthesis, boosting plant‐growth promoters. Compost amended with this iron oxide enhances crop growth, providing a sustainable strategy for upgrading waste into high‐value agricultural inputs.

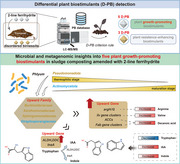

## 1 Introduction

The activated sludge process has been widely adopted in wastewater treatment plants (WWTPs), yet it generates vast volumes of waste‐activated sludge (WAS) as an inevitable by‐product.^[^ ^1^ ^]^ With the increasing demands of wastewater treatment,^[^ ^2^, ^3^ ^]^ annual WAS production in the United States, Europe, and China now exceeds 240 million tons.^[^ ^4^ ^]^ As a complex organic hemi‐solid in both organic resource and pollutant attributes, WAS presents a dual challenge: it must be treated safely to mitigate pollution risks, whileits potential as a recoverable resource remains largely underexploited.^[^ ^5^, ^6^ ^]^ Composting has long been regarded as a sustainable route for converting organic waste streams into value‐added fertilizers, offering a means to recycle nutrients and organic matter while improving soil health.^[^ ^7^, ^8^ ^]^ For WAS, composting is especially appealing as a low‐cost and environmentally friendly strategy for resource recovery. However, conventional composting of sludge often suffers from low product quality, as nearly half of the organic matter (OM) was lost as metabolic gases (e.g., CO_2_, NH_3_) through microbial mineralization.^[^ ^9^ ^]^ To improve compost quality and direct OM transformation toward humification, various strategies have been applied, including biodrying^[^ ^10^ ^]^ or hydrothermal pretreatment,^[^ ^11^ ^]^ and the addition of biochars,^[^ ^12^ ^]^ minerals,^[^ ^13^ ^]^ chemical agents,^[^ ^14^ ^]^ or microbial inoculants.^[^ ^15^ ^]^

Among these strategies, iron (Fe) and manganese (Mn) hydroxylated oxides have gained attention due to their large surface area and redox activity, which enhance humification and overall compost quality.^[^ ^16^ ^]^ Compared to their crystalline counterparts, disordered Fe and Mn hydroxylated oxides exhibit higher surface area and more reactive sites,^[^ ^17^ ^]^ facilitating the oxidation of precursors into quinone and semiquinone radicals and promoting humic substance formation.^[^ ^18^ ^]^ Additionally, their Lewis acid properties support condensation and nucleophilic addition reactions, yielding humification intermediates.^[^ ^19^ ^]^ However, compost maturity does not necessarily equal compost quality,^[^ ^20^ ^]^ and there is a growing shift toward compost evaluation based on chemical composition, particularly plant‐beneficial compounds, rather than traditional maturity indicators alone.^[^ ^20^ ^]^

Recent studies have revealed that sludge‐derived compost can serve as a source of plant biostimulants (PBs), including amino acids, allelochemicals, and phytohormones.^[^ ^14^, ^21^ ^]^ These compounds act as endogenous signaling molecules that enhance nutrient uptake, improve crop quality, and help mitigate stress in plants. The application of exogenous PBs has demonstrated clear benefits in agricultural settings. For instance, phytohormones regulate gene expression in stress‐response pathways,^[^ ^22^ ^]^ while amino acids like alanine can be directly absorbed by plants, bypassing nitrogen mineralization and conserving metabolic energy.^[^ ^23^ ^]^ In our previous work, polyferric sulfate addition during sludge dewatering enhanced humic acid and indoleacetic acid (IAA) production by 10.4% and 67.1%, respectively, through the stimulation of thermophilic bacterial activity.^[^ ^14^ ^]^ Despite the established role of Fe and Mn hydroxylated oxides in humification, their influence on PB biosynthesis, microbial metabolism, and downstream plant benefits during sludge composting remains poorly understood.

This study investigated the effect of two representative disordered Fe/Mn hydroxylated oxides (2‐line ferrihydrite and disordered birnessite) on sludge aerobic composting. The specific aims of this work were to: 1) evaluate their impact on key composting parameters (e.g., composting temperature, C/N ratio, pH, and OM content); 2) characterize PB composition and relative abundance via non‐targeted LC‐MS/MS using a custom PB database; 3) elucidate metabolic pathways and functional annotation of bacterial members associated with PB synthesis using time‐resolved metagenomics and genome‐resolved binning; 4) assess the agronomic performance of composts through plant growth experiments. These findings provide mechanistic insights into the role of Fe‐ and Mn‐based mineral amendments in promoting PB production and support composting strategies focused on maximizing product quality and fertility.

## 2 Materials and Methods

### 2.1 Mineral Synthesis and Characteristics

Ferrihydrite (2‐line) was synthesized following the method of Schwertmann and Cornell.^[^ ^24^ ^]^ In Brief, Fe(NO_3_)_3_·9H_2_O was gradually added to a KOH solution until the pH reached 7.0 ± 0.3. After allowing the precipitate to settle for 1–2 h, the supernatant was syphoned off. The precipitate slurry was then transferred to a beaker, immersed in 5L of ultrapure water, and left to settle. The overlying supernatant was then removed, and the beaker was refilled with ultrapure water. This washing process was repeated two or three times per day until the pH of the supernatant stabilized between 5 and 7 (generally 3–4 days). The final precipitate was then centrifuged at 3000 g for 10 min, and the supernatant was discarded.

Disordered birnessite was synthesized using the method of described by Villalobos.^[^ ^25^ ^]^ In brief, NaOH solution was slowly titrated with a KMnO_4_ solution over a maximum 5 min under vigorous stirring. Subsequently, a MnCl_2_·4H_2_O solution was added over a maximum of 30 min, also with vigorous stirring, resulting in the formation of a black precipitate. After settling for 4 h, the supernatant was syphoned off, and the remaining precipitate was centrifuged at 3000 g for 10 min, after which the supernatant was discarded. The residue was then shaken with 1 mol l^−1^ NaCl solution for 1 h and centrifuged. The NaCl washing step was repeated five times, with the final wash shaken overnight. The wash cycle was then repeated 10 additional times using ultrapure water in place of NaCl, until the supernatant pH stabilized at ≈12.8. Finally, the precipitate was dialyzed in ultrapure water using cellulose membrane tubing (molecular weight cutoff: 12000–14,000 g mol^−1^) until the conductivity of the external water dropped below 0.1 µS cm^−1^.

The synthesized minerals were stored as wet slurries at 4 °C. Their mineral identity and purity were confirmed by X‐ray diffraction (XRD) using a Bruker D8 Diffractometer with Cu‐Kα radiation. Diffractograms were recorded over a 2θ range from 10° to 80° (2θ) at a scan speed of 2°/min.

### 2.2 Feedstock Source and Composting Setup

A mixture of WAS and corn straw is used as the composting feedstock. The sourcing details of WAS and corn straw are provided in Table S1 (Supporting Information), while their basic physicochemical characteristics are presented in Table S2 (Supporting Information).

Composting experiments were conducted in a serial of 10L reactors, as described in our previous study.^[^ ^14^ ^]^ Briefly, 3 kg of dewatered sludge (≈80% moisture) was thoroughly mixed with 1.5 kg of corn straw to meet the moisture content and carbon‐to‐nitrogen ratio requirements for composting. To access the effects of 2‐line ferrihydrite and disordered birnessite on sludge composting, three treatment groups were established: traditional composting without mineral addition (TC), composting with 25 g 2‐line ferrihydrite (FeC, 5.5% [w/w‐feedstock]), and composting with 25 g disordered birnessite (MnC, 8.3% [w/w‐feedstock]). Intermittent Aeration was provided (10 min on/30 min off for the initial 7 days; 10 min on/50 min off thereafter).^[^ ^26^ ^]^ Composting samples were collected at day 0 (D0), day 5 (D5), day 10 (D10), day 15 (D15), day 20 (D20), day 25 (D25), and day 35 (D35), based on temporal temperature monitoring. Each compost pile was divided into five regions, from which subsamples (100 g per region) were collected at fixed locations and thoroughly homogenized. The mixed sample was then divided into two aliquots: one stored at 4 °C for physicochemical analyses, and the other another stored at −80 °C for biological analyses.

### 2.3 Chemical Analyses

The temperature was monitored using thermocouple probes. pH and electronic conductivity were determined from a 1:5 water‐soluble extract (w/v),^[^ ^27^ ^]^ which was also used for subsequent Dissolved organic matter (DOM) analyses. Dissolved organic carbon (DOC) was quantified with a TOC analyzer (TOC‐L, Shimadzu, Japan). Total carbon (TN) and total nitrogen (TN) contents were analyzed using an elemental analyzer (Vario MACRO CN Element analyzer). Sludge‐derived biopolymer concentrations were measured, with polysaccharide measured via the anthrone‐sulfuric acid method, using glucose as a standard.^[^ ^28^ ^]^ Protein and humic acid concentrations were analyzed using the Folin‐Lowry method, with Bovine Serum Albumin (BSA) and humic acid serving as standards.^[^ ^29^ ^]^ The fluorescence characteristics of DOM were determined by a 3D excitation‐emission matrix spectrometer (3D‐EEM, Hitachi F‐4600, Japan), and the corresponding fluorescence index (FIX), biological index (BIX), and humification index (HIX) were calculated using R (v4.2.2).^[^ ^30^ ^]^

### 2.4 PB Identification based on LC‐MS/MS

Generally, 2 g of the sludge sample was mixed with methanol/pure water (1:1 v/v) at a solid‐to‐liquid ratio of 1:5 (w/v) and sonicated for 20 min. After standing, the mixture was centrifuged at 3000 r min^−1^ for 15 min. The supernatant was transferred to a 5 mL volumetric flask and diluted to the mark with methanol. The solution was then filtered through a 0.22 µm micropore filter, and the filtrate was stored in a vial for LC−MS/MS analysis. Instrumental conditions were described in our previous study.^[^ ^14^ ^]^

The screening protocol for PB in compost and critical steps was constructed according to Cai,^[^ ^31^ ^]^ which includes systematic procedures such as data preprocessing, identification of diagnostic fragments, structural elucidation, and candidate prioritization. A comprehensive database of 456 PB compounds was constructed by integrating information from the literatures, metabolite repositories (KEGG, PubChem), and previous studies on plant biostimulants. The database includes phytohormones, amino acids and their derivatives, flavonoids, phenolic acids, and other secondary metabolites that have been experimentally validated or widely recognized for plant‐beneficial functions. These compounds have been reported to exhibit diverse plant‐beneficial functions, including growth promotion, biocontrol potential, stress resistance, and related activities. Compounds lacking sufficient evidence for a plant‐beneficial effect were excluded. The full list of compounds, together with references, is provided in the PB database (see SI Excel). The self‐built PB database and screening workflow were first imported into Compound Discoverer 3.3 (CD, Thermo Scientific). Raw LC‐MS/MS data, acquired in both positive and negative ion modes, were then imported into CD software. The screening criteria were as follows: retention time (RT) tolerance < 0.2 min, mass tolerance < 5 ppm, MS/MS matching scoring > 60%, minimum peak intensity > 50000 in negative ion modes and > 100000 in positive ion mode.^[^ ^31^ ^]^ Final identification of molecular structures was based on comparisons between the data‐dependent acquisition (DDA) MS^2^ spectra and RT between the candidate compounds and the PB database. Compound identification confidence was categorized according to the five‐level system proposed by Schymanski et al.,:^[^ ^32^ ^]^ level 1 (confirmed structure), level 2 (probable structure), level 3 (tentative candidates), level 4 (unequivocal molecular formula), and level 5 (exact mass of interest) (Figure S1, Supporting Information).

8 D‐PBs were quantitatively analyzed using HPLC‐grade external standards for the preparation of calibration curve samples. A primary stock solution of each analyte (1 g L^−1^) was prepared in a water/methanol (50:50, v/v) mixture. These stocks were subsequently serially diluted with a mixture of water/methanol (50:50, v/v) mixture to prepare working standard solutions spanning the expected D‐PBs concentration range in the biosolid samples. Calibration standard of each D‐PBs was prepared by spiking appropriate volumes of the working standard solutions into a blank matrix to generate a calibration curve. Then, the standards were subsequently analyzed by LC‐MS/MS under the same analytical conditions used for non‐targeted screening of PBs.

### 2.5 Metagenomic Analysis

A total of 27 temporal composting samples (*n* = 3 per time point, 5 g each) were preserved in 50% (v/v) ethanol and kept on ice during transportation. DNA was extracted using the E.Z.N.A. Stool DNA Kit (Omega Bio‐tek, Norcross, GA, USA) following the manufacturer's instructions. For each sample, 1 µg of genomic DNA was sheared using a Covaris S220 Focused‐ultrasonicator (Woburn, MA, America), and sequencing libraries were prepared with an average insert size of ≈450 bp. Paired‐end sequencing (2 × 150 bp) was performed on the Illumina NovaSeq 6000 platform.

Raw sequencing reads underwent quality trimming, assembly, binning, and refinement, resulting in 194 high‐ and medium‐quality metagenome‐assembled genomes (MAGs), based on MIMAG standards (completeness ≥ 80% and contamination ≤ 10%) (Text S1, Supporting Information). To ensure comparability across samples, gene abundances were normalized using the Transcripts Per Million (TPM). Taxonomic classification of MAGs was performed using GTDB‐TK (v2.2.6) against the GTDB r95 database. Taxonomies were then converted to the NCBI format with manual curation. Functional and metabolic potentials of each MAG was annotated using the METABOLIC pipeline with an integrated reference database. The relative abundance of each MAG were estimated as read coverage (%) using coverM (v0.6.1, https://github.com/wwood/CoverM), and genome copy numbers per million reads (CoPM) were calculated using MetaWRAP. Raw metagenomic sequence data have been deposited in the NCBI Sequence Read Archive under the accession number PRJNA1230542.

### 2.6 Pot Experiments

To evaluate the effects of 2‐line ferrihydrite and disordered birnessite on compost quality, a pot experiment was conducted using six different treatments in triplicate: i) Control (soil mixture only), ii) Ferrihydrite (soil mixture + 2‐line ferrihydrite), iii) Birnessite (soil mixture + disordered birnessite), iv) S‐TC (soil mixture + mature compost from TC), v) S‐FeC (soil mixture + mature compost from FeC) and vi) S‐MnC (soil mixture + mature compost from MnC). Each plastic pot (top diameter: 10 cm, bottom diameter: 7.2 cm, depth: 8.5 cm) was filled with a soil mixture consisting of 120 g of garden soil and 280 g of sand, moistened with deionized water to maintain a moisture content of ≈30%. The amount of mature compost added to each pot was standardized based on total organic carbon (TOC)^[^ ^33^ ^]^ with 2.09 g in S‐TC, 1.98 g in S‐FeC, and 1.38 g in S‐MnC. To evaluate the direct effects of mineral addition on plant growth, two treatments with only mineral additions (group “Ferrihydrite”: 0.145 g 2‐line ferrihydrite; group “Birnessite”: 0.101 g disordered birnessite) were included, corresponding to the Fe or Mn mineral contents in S‐FeC and S‐MnC treatments, respectively. Brassica rapa is one of the most widely cultivated leafy vegetables in Asia.^[^ ^34^ ^]^ Six *Brassi*ca.*rapa* seeds were evenly sown in each pot at a depth of ≈1 cm. Pots were irrigated regularly with deionized water. After 21 days, plant shoots were harvested and rinsed with deionized water. Root length, germination rate, fresh weight, and maximum leaf area (leaf length × width) were recorded for each pot. In addition, 100 g of rhizosphere soil was collected from each pot for analysis of biochemical properties, including ammonia and nitrate nitrogen levels, as well as rhizosphere microbial composition.

## 3 Results

### 3.1 Syntheses of Fe and Mn Hydroxylated Oxides

First, 2‐line ferrihydrite and disordered birnessite were synthesized before investigating their effects on sludge composting. 2‐line ferrihydrite and disordered birnessite are representative disordered Fe and Mn hydroxylated oxides, known for their poorly crystalline structure, higher surface area, and more reactive sites.^[^ ^17^ ^]^ These properties make them suitable for promoting OM stabilization and humification. As shown in XRD results (Figure S2, Supporting Information), the synthesized 2‐line ferrihydrite exhibits two broad diffraction peaks ≈2θ = 35° and 2θ = 62° (Figure S2A, Supporting Information). These two peaks are hallmark features of 2‐line ferrihydrite,^[^ ^35^ ^]^ that is the reason for the name of “2‐line”. For disordered birnessite, it is generally characterized by three board and diffuse peaks: one at 2θ = 12–13° (corresponding to the d‐spacing ≈7 Å), another at 2θ = 36–38° (d‐spacing ≈2.4 Å), and a third at 2θ = 65–66° (d‐spacing ≈1.4 Å).^[^ ^36^ ^]^ In our results, two peaks at 2θ = 36–38° and 2θ = 65–6° were detected, with the absence of 2θ = 12–13° (Figure S2B, Supporting Information). Accordingly, the peak at 2θ = 12–13° (001 basal reflection) represents the interlayer spacing between manganese oxide (MnO_2_) sheets and is a key feature of the layered structure of birnessite. The 2θ = 36–38° (100 reflection) represents in‐plane atomic ordering within the MnO_2_ layers, while 2θ = 65° (d‐spacing ≈1.4 Å) is associated with the Mn─O bond length within the layers.^[^ ^36^ ^]^ The absence of a peak at 2θ = 12–13° in our XRD spectrograms suggested the synthesized disordered birnessite is defective of layered structure. To conclude, it was confirmed that 2‐line ferrihydrite and disordered birnessite used in this study were successfully synthesized.

### 3.2 Effects of Fe and Mn Hydroxylated Oxide Additives on Composting Parameters and Biopolymer Functional Groups

**Figure 1:**
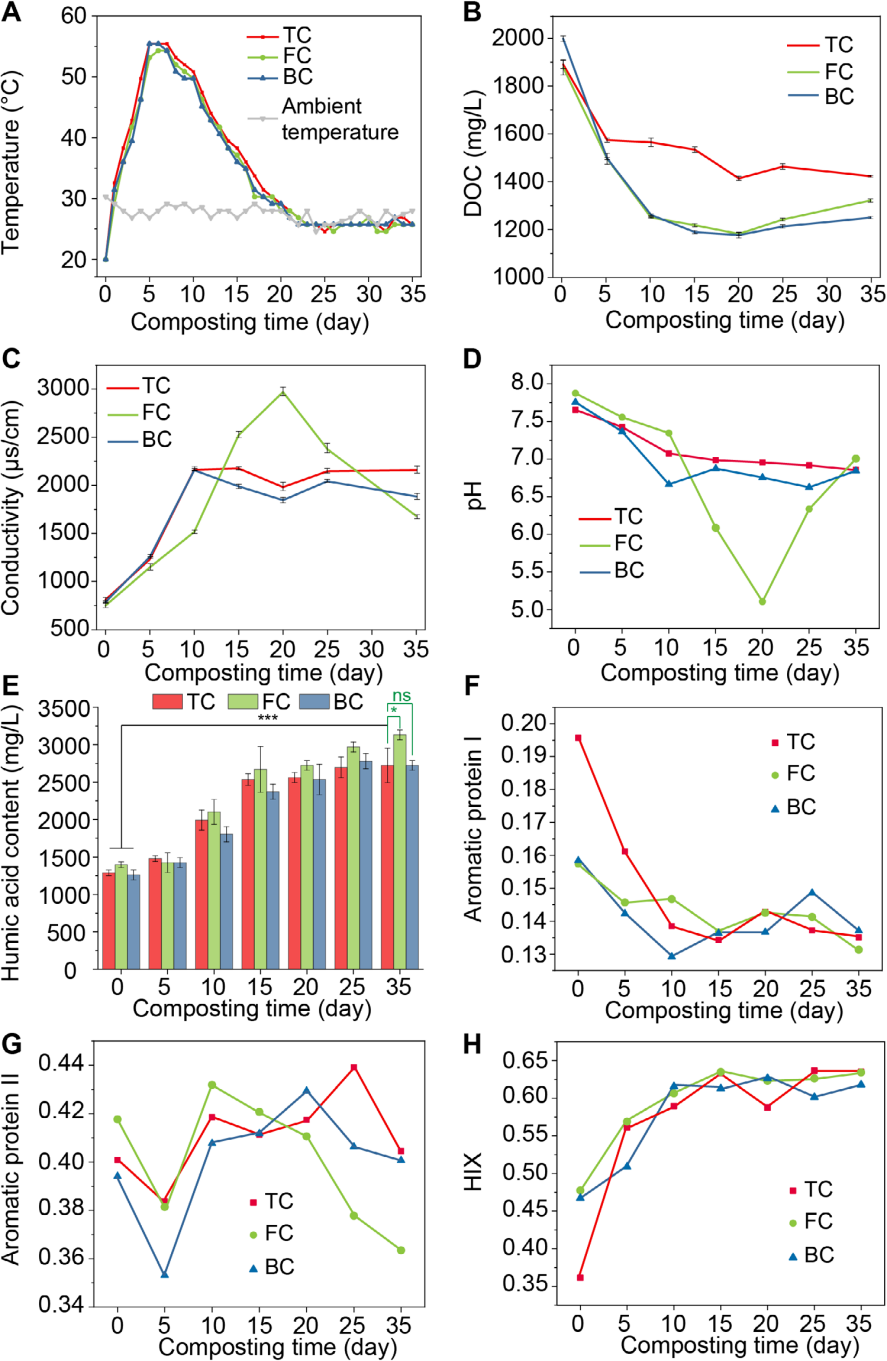
Dynamics of composting parameters and response of sludge‐based biopolymers in three composting treatments: traditional composting (TC), composting amended with 2‐lien ferrihydite (FeC), and composting amended with disordered birnessite (MnC). composting parameters including composting A) temperature, B) DOC, and C) conductivity, and D) pH. E) Humic acid concentration. Dynamics of F) aromatic protein I, G) aromatic protein II, and H) humification index (HIX) calculated from 3D‐EEM results.

As shown in **Figure** **1**A, the temperature in composting rosed from 20 °C to 55 °C in the first 5 days and remained above 50 °C until day 9 (thermophilic stage). Then, the temperature gradually declined to ambient (≈25 °C) by day 20 (cooling stage) and matured to end‐up compost until day 35 (maturation stage). Variations of carbon‐to‐nitrogen ratio (C/N) and moisture content also indicated that compost reached maturity on day 35 (Figure S3, Supporting Information). For the DOC value, it primarily declined at thermophilic and cooling stages, from 1878.1 (day 0) to 1411.1 mg L^−1^ (day 20) in TC. While in FeC and MnC, DOC decreased from 1878.1 mg L^−1^ (day 0, FeC) and 1999.0 mg L^−1^ (DO, MnC) to 1177.6 mg L^−1^ (day 20, FeC) and 1172.3 mg L^−1^ (day 20, MnC), respectively. An additional 11.9% (ΔDOC_FeC_/ΔDOC_TC_) and 15.9% (ΔDOC_MnC_/ΔDOC_TC_) DOC decline in FeC and MnC can be attributed to dissimilatory Fe(III) and Mn(IV) reduction, which drives DOC toward more chemical recalcitrant state.^[^ ^37^ ^]^ Fe(III) and Mn(IV) reducing bacteria such as *Shewanella* and *Geobacter* are able to mediate electron transfer to insoluble Fe(III) and Mn(IV) oxides via direct or indirect contacts, where microorganisms attach directly to mineral surfaces or rely on electron shuttles, e.g., aromatic/quinone compounds in humic OM.^[^ ^38^ ^]^ Conductivity, which reflects the concentration of dissolved ions (e.g., salts, minerals, and metals), showed a similar trend in both TC and MnC treatments—increasing during the first 10 days and then remaining relatively stable until the end of the composting process. In contrast, conductivity in FeC continued to rise by D20, resulting in an extra 36.7% and 37.7% increase relative to TC (*p*< 0.05, *t*‐test) and MnC (*p*< 0.05, *t*‐test), respectively. The reason behind this increase can be attributed to the hydrolysis of ions and an increase of free protons after 2‐line ferrihydrite addition, which resulted in an observed decline in pH to 5.11 and facilitated the release of ions during composting. Protein, polysaccharide, and humic substances are the primary biopolymers, accounting for 70–90% of the total DOM in sludge.^[^ ^39^ ^]^ As shown in Figure 1b, FC generated 14.7% more humic substances (HS) than TC (*p*< 0.05, *t*‐test) and MnC (*p*< 0.01, *t*‐test), accompanied by a decrease in protein and polysaccharide content (R^2^ = −0.716, *p *> 0.05) (Figure S4, Supporting Information). In the results of 3D‐EEM (Figure 1C), three composting groups consumed an equal amount of aromatic protein I (e.g., tryptophan‐like), while FeC utilized more protein II (e.g., tyrosine‐like) than TC and MnC.

According to previous reports, Fe oxides promoted humification degree through increasing the relative abundance of thermophilic microorganisms,^[^ ^40^, ^41^ ^]^ and Mn minerals enhanced HS formation via abiotic catalysis.^[^ ^42^, ^43^, ^44^ ^]^ In this study, MnC exhibited HS content comparable to that of TC, suggesting that the biotic composting process may have inhibited abiotic HS catalysis. This inhibition could be attributed to microbial metabolism and the accumulation of organic intermediates that compete with or interfere in Mn redox reactions, thereby masking the catalytic role of disordered birnessite.^[^ ^45^, ^46^ ^]^ In particular, Mn‐reducing microorganisms may lower the oxidative capacity of Mn oxides, while organic acids produced during composting could complex with Mn minerals, both of which diminish their abiotic catalytic contribution to humification.^[^ ^47^, ^48^ ^]^

### 3.3 Differential PB Identification During Sludge Composting in Response to Fe or Mn Hydroxylated Oxides Addition

**Figure 2:**
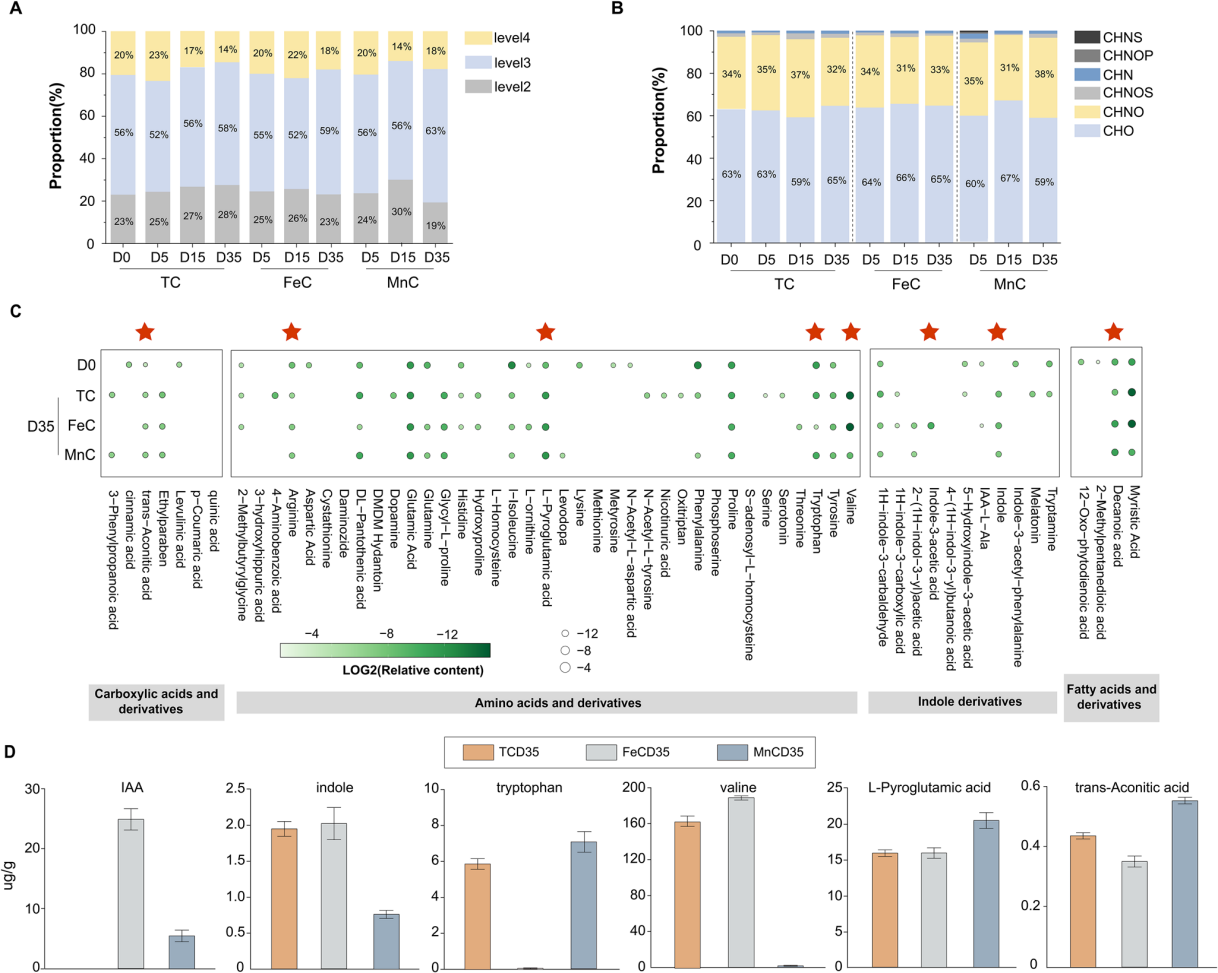
Profiles of PBs in TC, FeC and MnC composting groups. A) Molecular identification levels and B) element combinations in three composting samples. C) Profile of PBs detected in samples. Red stars and red fonts indicate D‐PBs between FeC/MnC and TC. D) quantification analysis of D‐PB.

To detect potential PB profiles in different composting groups, a self‐built PB database integrating a non‐targeted screening workflow was utilized to identify PB, as depicted in Figure S5 (Supporting Information). Among all groups, 52–63% of identified PB molecules belonged to level 3, followed by 19–30% in level 2 and 14–23% in level 4 (**Figure** **2**A). The elemental combinations of PB molecules primarily consisted of C_4‐30_H_6‐48_O_2‐16_ and C_3‐21_H_7‐19_N_1‐5_O_1‐7_, accounting for 59–67% and 30–37% of the total PB molecules, respectively (Figure 2B). In the mature compost, a total of 136 PB molecules were identified, with 14.7% (20) molecules and 8.8% (12) molecules exclusively belonging to FC and BC groups, respectively (Figure S6, Supporting Information). Then, we investigated the changes of PB in different groups across different composting stages (Figure S7, Supporting Information). Among identified PBs, amino acids and their derivatives increased during the thermophilic stage and then declined in the cooling and maturation stages. Indole and its derivatives continuously increased during composting. Other PB categories such as terpenoids, fatty acids, and their derivatives displayed varying trends across different groups. In TC, their content presented a continuous upward trend, while in the MnC and FeC groups, it peaked during the cooling stage before declining in the maturation stage.

To better understand the effect of Fe or Mn hydroxylated oxides, differential PBs between mineral‐amended groups (FeC and MnC) and TC were further analyzed (Figure 2C), with PB identification standards detailed in Table S3 (Supporting Information). Overall, compared to the control (TC), the FeC treatment primarily elevated the levels of valine (FeC_D35_ = 188.607 ug/g), indole (FeC_D35_ = 2.02 ug g^−1^), and IAA (FeC_D35_ = 24.89 ug g^−1^) (Figure 2D). In contrast, the MnC treatment specifically increased the relative content of tryptophan (MnC_D35_ = 7.06 ug g^−1^), L‐pyroglutamic acid (MnC_D35_ = 20.45 ug g^−1^), and trans‐aconitic acid (MnC_D35_ = 0.56 ug g^−1^). Both treatments led to a moderate increase in arginine (FeC_D35_ = 1.61 ug g^−1^, MnC_D35_ = 1.55 ug g^−1^) and decanoic acid (FeC_D35_ = 8.29 ug g^−1^, MnC_D35_ = 8.28 ug g^−1^). However, a notable suppressive effect was observed for several other amino acids, including proline, tyrosine, isoleucine, and glutamic acid, which were significantly lower in both the FeC and MnC groups. According to the previous work, the reduction of these amino acids might contribute to amino acid bio‐conversion to indoles, IAA, and other plant hormones or synthetic PB intermediates.^[^ ^49^ ^]^

### 3.4 The Metagenomic Insights into Differential PB Production During Sludge Composting in Response to Fe‐ and Mn Hydroxylated Oxides Additions

**Figure 3:**
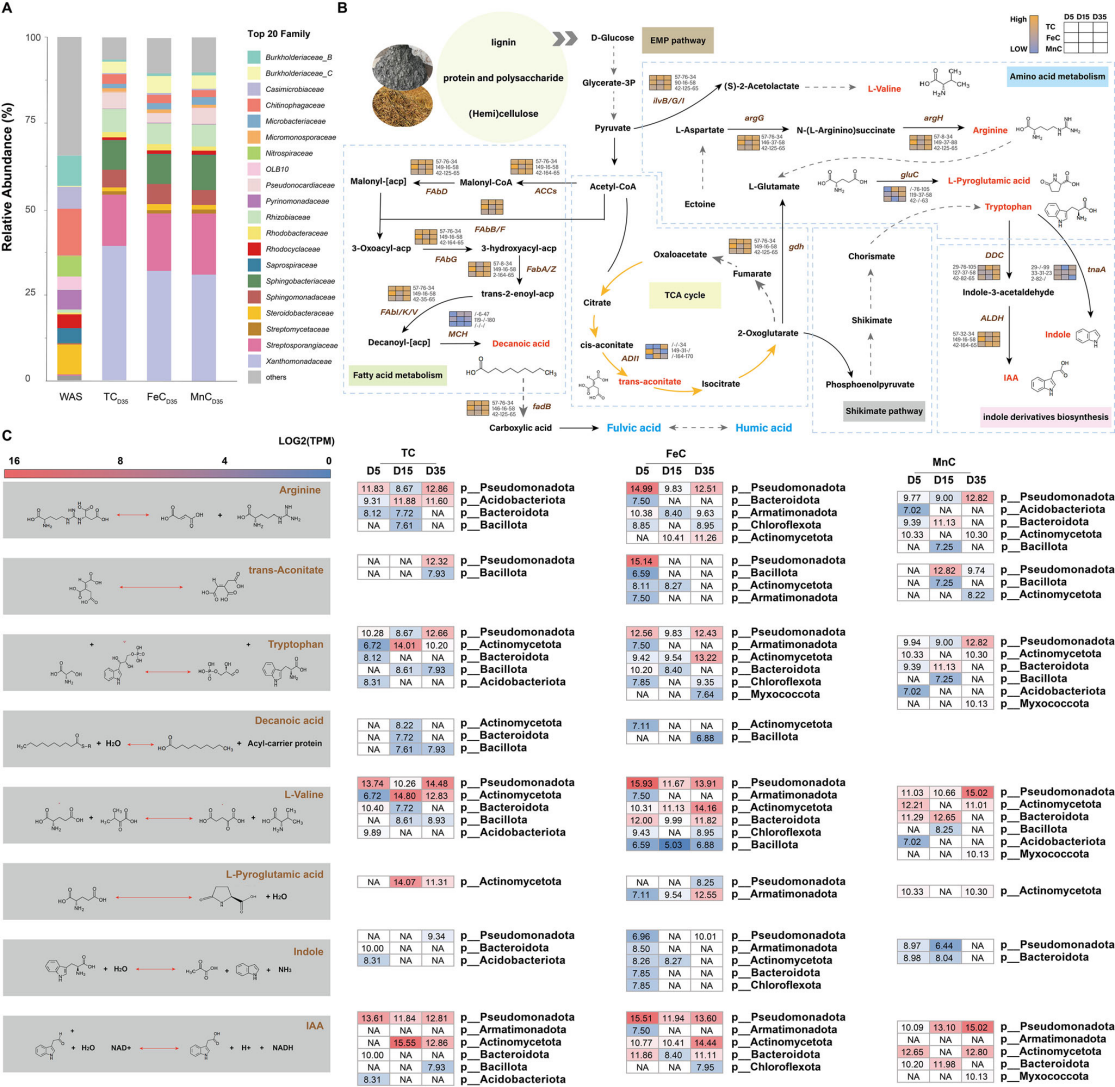
Metabolic pathway and taxonomic annotation of differential PBs (D‐PBs) in FeC, MnC, and TC composting groups. A) Microbial abundance information of the top 20 families. B) D‐PB synthesis pathways and related metagenomic information. C) Microbial abundance in the final step of D‐PB synthesis. The red font indicates D‐PB and the brown italics represent genes that regulate this pathway. The gray dashed lines represent omitted pathway information, with details shown in Figure S9 (Supporting Information).

Eight upwardly differential PBs (D‐PB) were identified in response to Fe or Mn hydroxylated oxides addition, including Valine, Tryptophan, Arginine, L‐Pyroglutamic acid, Indole, Trans‐Aconitic acid, IAA, and Decanoic acid. Further, we reconstructed the metabolic pathways of D‐PBs assembled with taxonomic and genetic information, to understand the microbial mechanisms behind the production of these D‐PB (**Figure** **3**). Compared to the WAS group, microbial community structure underwent significant changes during maturation, characterized by increased relative abundances of *Xanthomonadaceae* and *Sphingobacteriaceae*, whereas differences among the matured compost groups were relatively minor (Figure 3A). The organic fraction in the initial feedstock serves as the main source for D‐PB production. In detail, hydrolysates of lignin, cellulose in corn straw, and polysaccharides in WAS can be converted into pyruvate via glycolysis,^[^ ^50^ ^]^ then enter the tricarboxylic acid (TCA) cycle for the synthesis of PBs.^[^ ^51^ ^]^ As depicted in Figure 3B, valine, arginine, and L‐pyroglutamic acid were synthesized via amino acid metabolism through the Embden‐Meyerhof‐Parnas (EMP) pathway, which supplies the necessary carbon skeletons and energy required for amino acid synthesis. Glutamic acid, the precursor of L‐pyroglutamic acid, can be generated through arginine metabolism or transamination of 2‐oxoglutarate in the TCA cycle, and the synthesis of tryptophan relies on the cooperative action of the TCA cycle and the shikimic acid pathway. Meanwhile, the TCA cycle provides both energy and intermediate products that serve as starting materials for the shikimic acid pathway, thereby facilitating the completion of tryptophan synthesis.^[^ ^51^ ^]^ Then, indole and IAA were subsequently synthesized with tryptophan as a precursor. Trans‐Aconitic acid serves as an intermediate in the TCA cycle. While decanoic acid is produced through the fatty acid metabolic pathway, which is interconnected with the TCA cycle to provide essential precursors for its synthesis. In the taxonomic annotation of D‐PB‐related pathways, the phyla *Pseudomonadota* and *Actinomycetota* dominated D‐PB production in FeC and TC, while in MnC, PBs were mainly generated by the phyla *Actinomycetota*, *Bacteroidota*, and *Pseudomonadota*. Though D‐PB‐mediated microorganisms at the phylum level were similar between TC and FeC, their microbial compositions and PB conditions showed significant differences according to the D‐PB conditions described above and assembled bin information in TC (bin57, 76, and 43) and MnC (bin146, 149, 37, and 58).

According to Figure 3C, we observed that the primary differences in the dominant microbes lie in the final step of D‐PB synthesis. Therefore, taxonomic annotation and genetic information related to the final step of each D‐PB were analyzed, as shown in Figure 3C. Notably, FeC enhanced the abundance of *Pseudomonadota* during the thermophilic phase (Figure 3B,C). In addition, the relative abundance of *Actinomycetota* gradually increased in FeC along the synthesis pathways of L‐pyroglutamic acid, tryptophan, valine and, IAA (Figure 3C). In the final step of most D‐PB synthesis (valine, arginine, tryptophan, and IAA), a greater diversity of microbial species was observed at the phylum level in FeC compared to MnC and TC during maturation. This observation aligns with the increased relative abundance of valine, arginine, and IAA during the maturation stage, in contrast to the decreased levels of tryptophan and the stable abundance of L‐pyroglutamate in FeC (Figure 2D).

**Figure 4:**
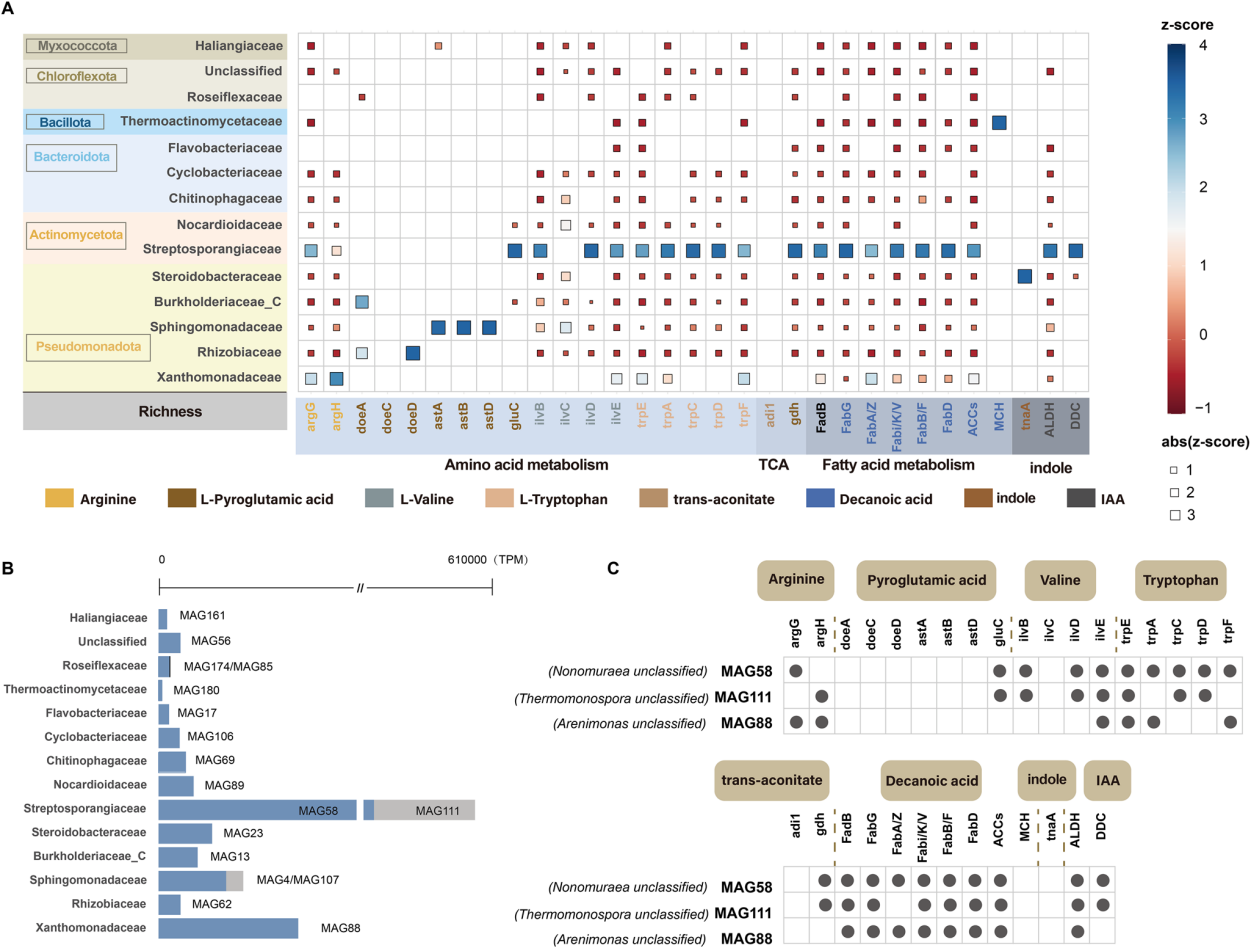
Functional annotation and gene information that regulate the synthesis of eight D‐PB in FeCD35. A) Taxonomic annotation and gene information of D‐PB synthesis regulation. B) Microbial classification annotation of MAG related to D‐PB synthesis. C) The top three MAGs involved in regulating D‐PB.

As concluded, 2‐line ferrihydrite can promote PB production during sludge composting. To clarify the reason for enhanced D‐PB condition in FeC, more details in D‐PB related genes and the microorganisms at family level were conducted. In particular, members of the family *Streptosporangiaceae* within the phylum *Actinomycetota* were strongly associated with the biosynthesis of multiple PBs, notably enhancing the levels of IAA and tryptophan synthesis genes (*trpA/C/D/E/F*, *ALDH, DDC*). During the maturation stage of composting, the relative content of tryptophan decreased, while the concentrations of indole and IAA increased. This was accompanied by an expansion in microbial diversity linked to tryptophan synthesis and upregulation of *trpA/C/D/E/F*, *ALDH*, and *DDC*, suggesting that 2‐line ferrihydrite addition may reflect enhanced conversion of tryptophan to indole and IAA. Meanwhile, the genes related to arginine and valine synthesis, namely *argH/G* and *ilvB/C/D/E*, also exhibited a significant upward trend in *Streptosporangiaceae*, *Xanthomonadaceae*, and *Sphingomonadaceae*. In the biosynthetic pathway of decanoic acid, *Fab* gene clusters and *ACCs* genes that regulate carbon chain elongation are predominantly upregulated by *Streptosporangiaceae* and *Xanthomonadaceae*. Additionally, the *MCH* gene, responsible for regulating the hydrolysis process involved in decanoic acid production, is specifically upregulated by *Thermoactinomycetes*. These findings align with the above conclusions that FeC increased in the relative concentrations of arginine, valine, and decanoic acid. However, the observed upregulation of the key synthetic gene *gluC* for L‐pyroglutamic acid in *Streptosporangiaceae*, along with the gradual increase in the abundance of *Actinomycetota*, differs from the conclusion that the relative content of it remained nearly constant during the maturation stage (Figure 2D, **Figure** **4**).

To assess the genomic representation of the dominant microbial families identified in Figure 4A and to clarify their specific functional contributions, metagenomic analysis was conducted. This analysis resulted in the reconstruction of 194 high‐quality metagenome‐assembled genomes (MAGs). Functional annotation of these MAGs revealed 17 genomes encoding genes associated with D‐PB synthesis (Figure 4B). Among them, members of the family *Streptosporangiaceae* (MAG58, MAG111) exhibited the highest TPM values, followed by *Xanthomonadaceae* (MAG88). According to the functional annotation results (Figure 4C), these two families exhibited similar metabolic profiles, indicating potential synergistic activity within shared biosynthetic pathways. Specifically, MAG58 (*Nonomuraea* unclassified), MAG111 (*Thermomonospora* unclassified), and MAG88 (*Arenimonas* unclassified) were primarily enriched in genes responsible for the biosynthesis of arginine, valine, tryptophan, decanoic acid, and IAA.

In summary, *Pseudomonadota* and *Actinomycetota* dominate D‐PB biosynthetic pathways in FeC, with *Pseudomonadota* showing a significant increase in abundance during the thermophilic stage. The addition of 2‐line ferrihydrite exhibited higher microbial diversity and enriched gene abundances (*trpA/C/D/E/F*, *argH/G*, *ilvB/C/D/E*, *ACCs*, and *Fab* gene clusters) carried by *Xanthomonadaceae*, *Sphingomonadaceae* and *Streptosporangiaceae*, which correlated with higher levels of arginine, valine, and decanoic acid. Furthermore, FeC enhanced the conversion of tryptophan to IAA and indole by enriching gene abundances related to *ALDH*, *DDC*, and *tnaA*, which was reflected in the corresponding increase in these compounds.

### 3.5 Effect of Fe and Mn Hydroxylated Oxides Additives on Compost Fertility

**Figure 5:**
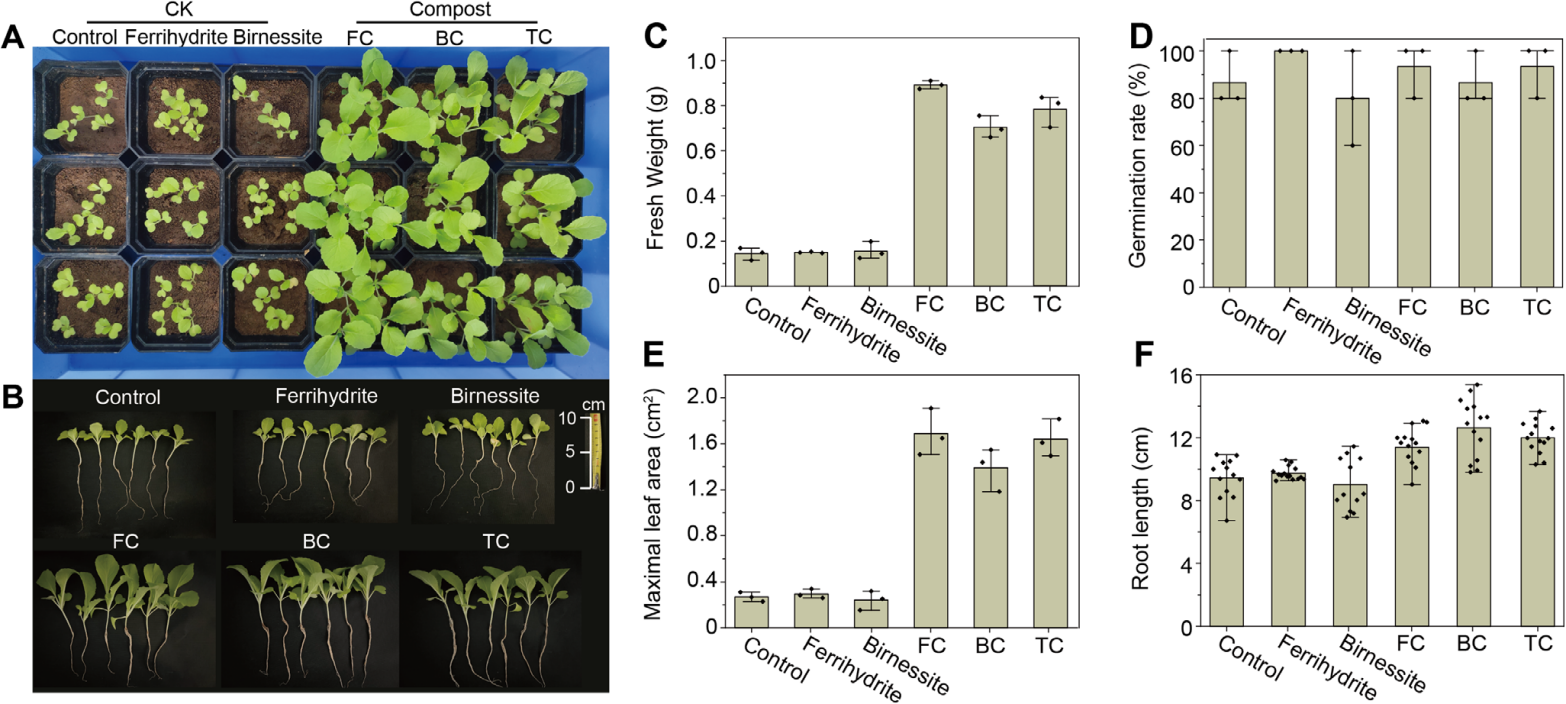
Fertilization evaluation of mature compost on plant growth under the exposure of the mature composts in TC, FeC, and MnC treatments. A) Overall plant photograph. B) growth photograph in representative pots. C) Fresh weight, D) germination rate, E) leaf area, and F) Root length of *Brassica rapa* seeds after 21d cultivation.

To assess the effect of 2‐line ferrihydrite and disordered birnessite additives on compost quality, pot experiments treated with mature composts in TC, MnC, and FeC groups were carried out in triplicate. After 21 days of cultivation, plant phenotypes, rhizosphere microorganisms, and nutrient concentration in pots were analyzed. In CK pots without compost addition (Control), as well as pots with only 2‐line ferrihydrite (Ferrihydrite) and disordered birnessite (Birnessite) additions, plants exhibited limited growth than compost groups. This demonstrated that neither ferrihydrite nor birnessite could directly promote plant growth, underscoring the plant growth‐promoting effects of sludge compost. Fresh weight is the most straightforward index indicating compost fertility. In composting groups, this value exhibited an order of S‐FeC (0.89 g) > S‐TC (0.78 g) > S‐MnC (0.70 g) (**Figure** **5**C), which was consistent with the photograph in Figure 5A,B. Therein, the average fresh weights of harvested plants treated with FeC and MnC increased by 14.1% (*p*< 0.05, *t*‐test) and −8.9% (*P*< 0.001, *t*‐test), compared to TC. Combined with similar inorganic nutrient (nitrogen) assimilation by plants in S‐TC and S‐FeC groups (Figure S10, Supporting Information), the superior fertilization performance of S‐TC may be attributed to the promoted PB conditions after 2‐line ferrihydrite addition. Meanwhile, the reduced nitrate content in S‐BC during the seeding stage led to inhibited nitrate assimilation by plants, which may be a key factor contributing to its decreased fresh weights. Maximal leaf area is a key index that reflects plant health conditions and environmental adaptability. The values of S‐FeC (1.69 cm^2^) and S‐MnC (1.38 cm^2^) were 3.0% (*p *> 0.05, *t*‐test) and −15.8% (*p* < 0.05, *t*‐test) higher in relative to TC (1.64 cm^2^) (Figure 5E). This implies that FeC had no significant differences with TC on plant maximal leaf area, while MnC had an inhibitory effect. As for root length (Figure 5F), S‐MnC had the highest average value of 12.6 cm, followed by S‐TC (12.0 cm) and S‐FeC (11.4 cm). The relatively higher root length observed in S‐MnC can be caused by limited nutrients or environmental stress, as root elongation improves plant viability (harvest stage) in constrained environments. The rhizosphere is a narrow zone possessing high microbial diversity and activity with feedback effects on root development and plant growth. PCA analysis showed that the rhizosphere bacteria profile of S‐FeC and garden soil were similar, while both differed significantly from those of S‐MnC and S‐TC groups (Figure S11, Supporting Information). In detail, S‐FeC increased the relative abundance of phyla *Pseudomonadota* and *Bacteroidota, and Actinomycetota* in rhizosphere (Figure S11, Supporting Information), in relative to S‐TC. At phylum level, S‐FeC mainly increased the level of *Lysobacter*, *Ramlibacter*, and *Sphingomonas*. As concluded, it was believed that PB triggered by 2‐line ferrihydrite additives contributed to the enhanced fertilization performance of mature compost.

## 4 Discussion

### 4.1 D‐PB Promotes Plant Growth in Various Aspects

In this study, 2‐line ferrihydrite has been demonstrated to enhance the generation of several PBs in the end‐up compost, including amino acids (valine, arginine), fatty acid (decanoic acid), and indole derivatives (indole, IAA), while disordered birnessite promoted the production of amino acids (tryptophan, arginine, L‐pyroglutamate), carboxylic acids (trans – aconitic acid) and fatty acid (decanoic acid).

For D‐PB with increased relative content in the FeC, IAA, a key plant hormone, regulates various aspects of plant growth, including cell elongation, root development, and apical dominance.^[^ ^52^ ^]^ It has been reported that indoles exhibit allelopathic effects and act as interkingdom and interspecies signaling molecules, inhibiting the growth of certain competitive plants while promoting the proliferation of beneficial rhizosphere microorganisms.^[^ ^53^ ^]^ For amino acid‐based PBs, valine, tryptophan, and arginine play a crucial role in protein stress responses and growth regulation.^[^ ^54^ ^]^ In addition, valine positively impacts the accumulation of carbon and nitrogen nutrients and contributes to increased lignin.^[^ ^55^ ^]^ Regarding D‐PB with increased relative content in the MnC, tryptophan serves as an important precursor for various metabolites, including auxin. L‐pyroglutamate has been shown to enhance plant stress resistance, such as drought tolerance,^[^ ^56^ ^]^ while trans‐aconitic acid exhibits promising effects on the control of agricultural insect diseases.^[^ ^57^ ^]^ Decanoic acid increased in both FeC and MnC in the mature compost, it can benefit in stress resilience^[^ ^58^ ^]^ and growth promotion of plants through related compounds or derivatives.^[^ ^58^, ^59^ ^]^ Arginine, a precursor for the biosynthesis of important messengers such as polyamines and nitric oxide (NO), plays a pivotal role in almost all physiological and biochemical processes in plants.^[^ ^60^ ^]^ Notably, L‐pyroglutamic acid and trans‐aconitic acid, which exhibit increased relative content following the addition of birnessite, appear to focus more on enhancing plant stress resistance. In contrast, IAA, indole, and valine, promoted by the addition of 2‐line ferrihydrite, seem to be more involved in regulating plant growth. These findings are consistent with the results of the pot experiment, where 2‐line ferrihydrite addition increased plant growth (fresh weight increased by 14.1%, average leaf area increased by 3%) compared to TC, while MnC had no promotion effects on these indexes.

### 4.2 The Potential Microbial Mechanism of PB Production Affected by 2‐Line Ferrihydrite Addition

**Figure 6:**
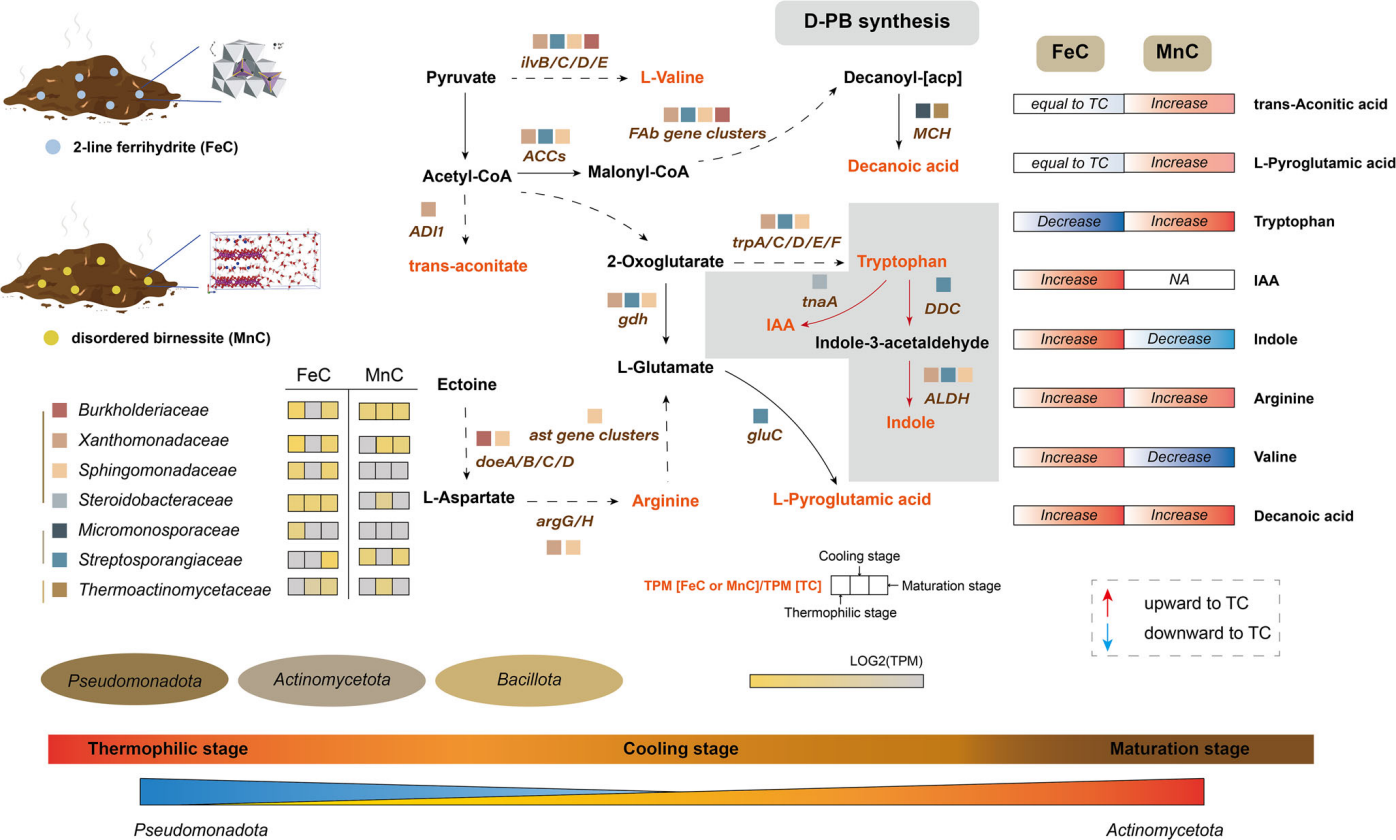
Overall transformation of D‐PB synthesis in sludge composting amended with 2‐line ferrihydrite and disordered birnessite.

*Pseudomonadota* and *Actinomycetota* were the primary contributors to the final step of D‐PB biosynthetic pathways, with *Pseudomonadota* exhibiting a marked increase in abundance during the thermophilic phase. The abundance levels of *argH/G*, *ilvB/C/D/E*, *ACCs*, and *Fab* gene clusters carried by *Xanthobacteriaceae*, *Sphingobacteriaceae*, and *Streptobacteriaceae* increased upon the addition of 2‐line ferrihydrite, which in turn promoted the biosynthesis of arginine, valine, and decanoic acid. Concurrently, the increased abundance of *ALDH*, *DDC*, and *tnaA* might have facilitated the conversion of tryptophan into IAA and indole (**Figure** **6**).

The abundance of microorganisms harbouring these genes varied across the stages of FeC and MnC. Specifically, *Xanthobacteriaceae*, which played a significant role in synthesizing most D‐PB in FeC, particularly valine and tryptophan, were predominantly observed during the thermophilic and maturation stages of FeC. In contrast, in MnC, *Xanthobacteriaceae* appeared during the cooling and maturation stages, with higher abundance noted in FeC. This observation partially elucidates the increase in valine during the maturation stage, consistent with previous results in FeC. Additionally, *Steroidobacteraceae*, which regulates the indole‐synthesizing gene *tnaA*, exhibited high abundance throughout all stages of FeC, whereas in MnC, it was only present during the cooling stage, consistent with the observed decline in the relative content of indole in MnC (Figure 6).

Our metagenomic data reveal a correlation between ferrihydrite addition, microbial abundance, and functional gene upregulation, but the exact mechanistic link cannot be fully resolved in this study. Previous reports suggest that ferrihydrite may act both directly, as a redox‐active mineral utilized by Fe‐reducing microorganisms (e.g., *Pseudomonadota* and *Actinomycetota*),^[^ ^61^, ^62^ ^]^ and indirectly, by altering local redox potential, micronutrient availability, or organic matter interactions.^[^ ^63^ ^]^ It is therefore plausible that the observed microbial and functional responses reflect a combination of these processes, although further experiments would be needed to disentangle their relative contributions. In addition to the influence on the abundance of related synthetic genes, 2‐line ferrihydrite may also catalyze the oxidation of tryptophan to IAA and indole by generating hydroxyl radicals.^[^ ^64^ ^]^ In FeC group, both the microbial species and gene abundance level in the L‐pyroglutamic acid synthesis pathway showed an upward trend while its relative content remained approximately constant compared to TC. One possible explanation is the strong affinity of L‐pyroglutamic acid, owing to its high carboxyl density, for ferrihydrite nanoparticles (FNPs), which may reduce its bioavailability and detectable content in the composting system.^[^ ^65^ ^]^ Alternatively, the discrepancy may reflect the rapid utilization of L‐pyroglutamic acid as a metabolic intermediate, for instance being funneled into glutamine biosynthesis or the TCA cycle, which prevents its accumulation.^[^ ^66^, ^67^ ^]^ In addition, the elevated temperature and oxidative conditions during composting could promote chemical transformation or integration of this compound into humified organic matter,^[^ ^68^ ^]^ thereby limiting its detectable concentration despite increased microbial and genetic potential for its synthesis. Despite valine and L‐pyroglutamate exhibiting comparable carboxyl group densities, the relative abundance of valine is elevated owing to the greater variety of microorganisms participating in the final step of valine biosynthesis and the higher expression levels of the *ilvB/C/D/E* genes implicated in this process (Figure 3B). In addition, studies have shown that lignin and carboxylic‐rich alicyclic molecules (CRAM) can undergo decomposition during composting, releasing nitrogen‐containing substances that are essential for synthesizing amino acids like arginine and valine.^[^ ^69^ ^]^ Additionally, in the final step of D‐PB synthesis, 2‐line ferrihydrite promotes an increase in the abundance of *Pseudomonadota* and *Actinomycetota*, which play significant roles in lignin degradation through the production of lignin‐degrading enzymes and metabolic processes.^[^ ^70^, ^71^ ^]^ This enhancement aids in the conversion of lignocellulosic biomass into precursor compounds, thereby facilitating certain PB synthesis.^[^ ^72^ ^]^

It should be noted that some pathways of D‐PB synthesis are still not clear yet, especially the enzymes involved in the final hydrolysis step of decanoic acid synthesis, which warrants further study. In addition, the potential resistance‐enhancing effect inferred for the MnC group was mainly based on molecular evidence (e.g., the accumulation of L‐pyroglutamate and trans‐aconitic acid), and future long‐term or field‐scale experiments under environmental stress conditions are needed to verify this effect. Beyond mechanistic insights, it is also important to consider the broader implications of applying Fe/Mn‐amended composts in agricultural soils. Owing to their strong sorption capacity and redox activity, these minerals may influence soil redox chemistry and trace element dynamics. While the relatively low dosage used in this study suggests that such effects are minor under our conditions, the long‐term environmental outcomes remain to be systematically evaluated. Future field‐scale research should therefore integrate agronomic performance with monitoring of soil redox potential, trace metal speciation, and microbial community stability to ensure sustainable application.

## Conflict of Interest

The authors declare no conflict of interest.

The data that support the findings of this study are available from the corresponding author upon reasonable request.

## Supporting information



Supporting Information

Supplemental Data
