# Supplementary material for: 2‐line Ferrihydrite Enhance Microbial Synthesis of Plant Biostimulants in Composted Biosolid by Regulating Phyla Pseudomonadota and Actinomycetota
Source: Adv Sci (Weinh). 2025 Dec 1;13(9):e06502. doi: 10.1002/advs.202506502 (PMC12904056; doi:10.1002/advs.202506502)
Supplement: Supplementary file 1 — Supporting Information [file ADVS-13-e06502-s002.docx]

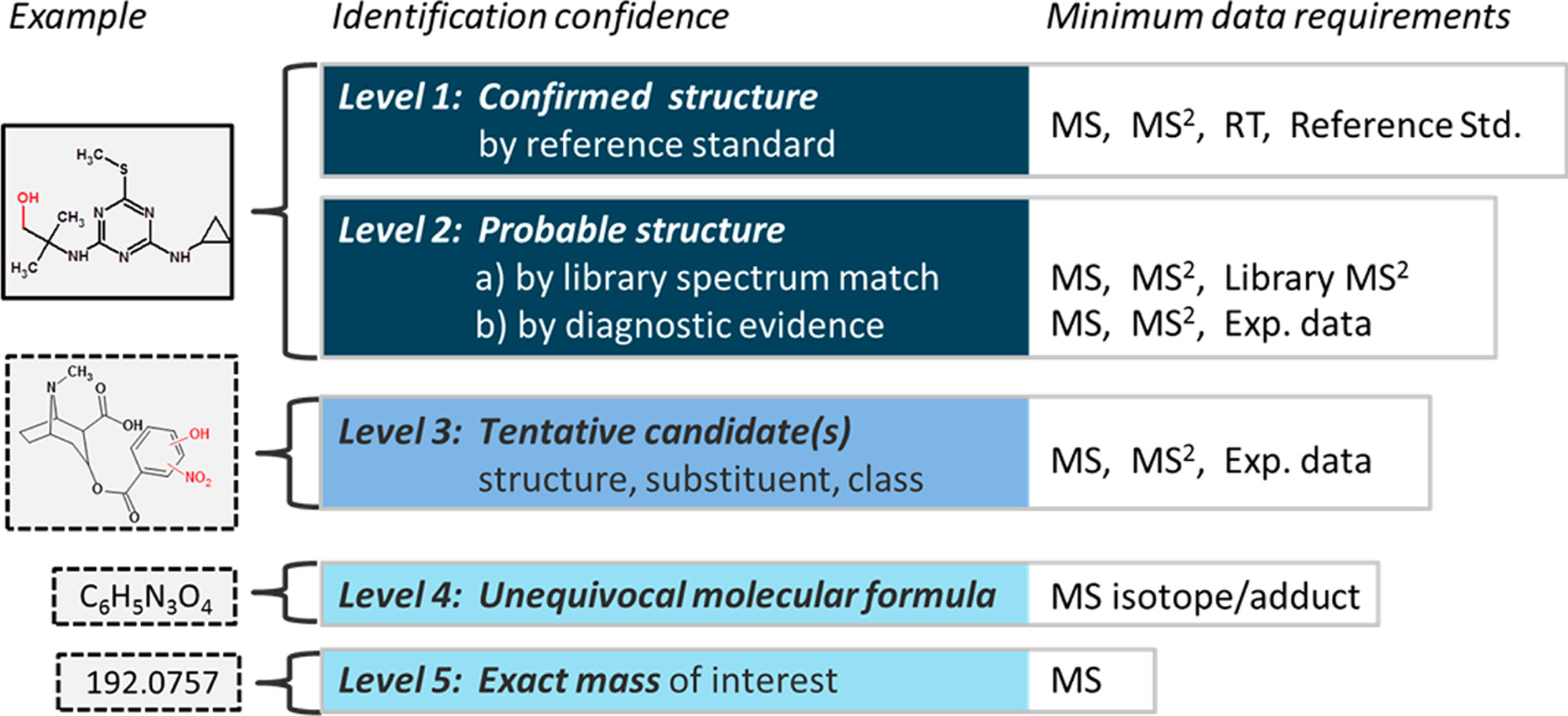


**Fig S1** Proposed identification confidence levels in high resolution mass spectrometric analysis. Note: MS2 is intended to also represent any form of MS fragmentation^36^.


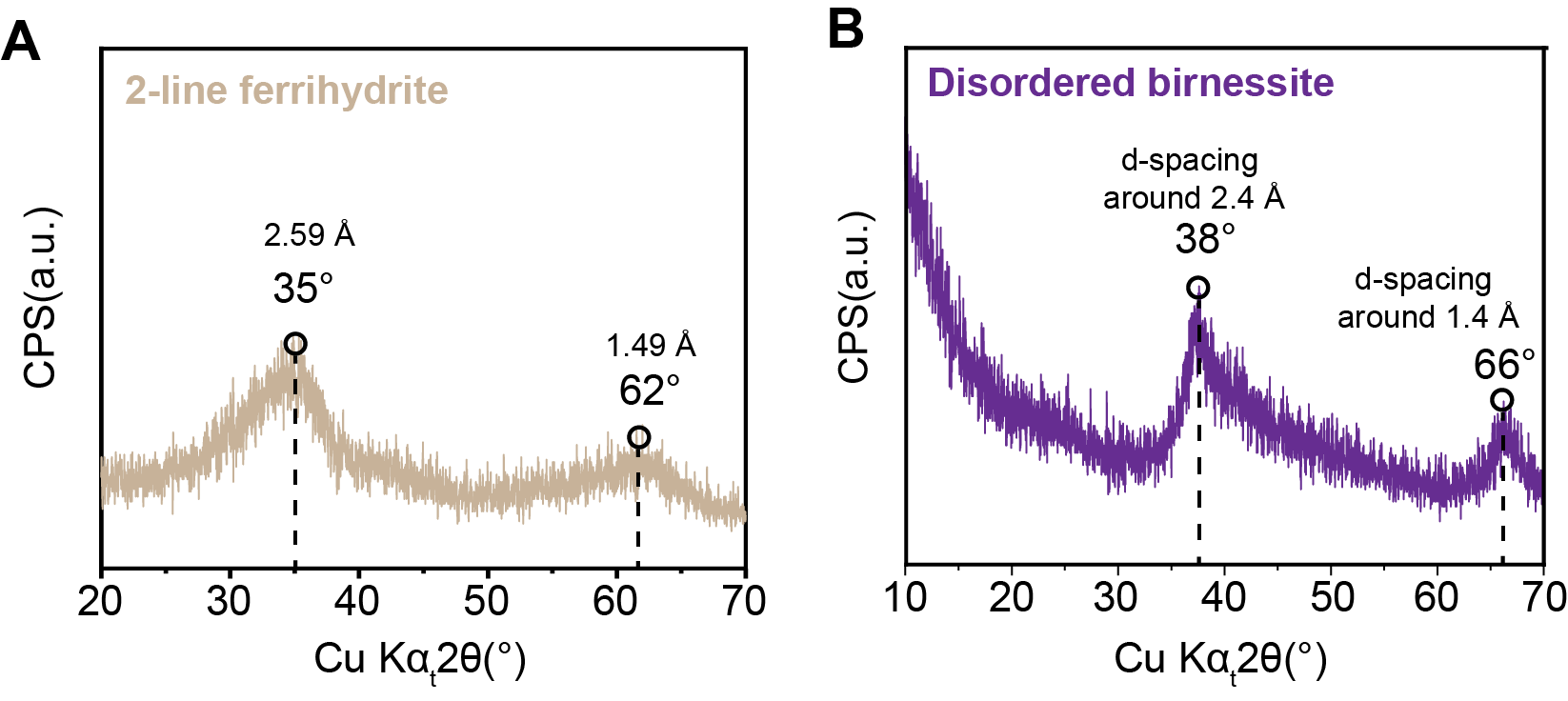


**Fig S2** X-ray diffraction spectrogram of synthesized (A) 2-line ferrihydrite and (B) disordered birnessite.


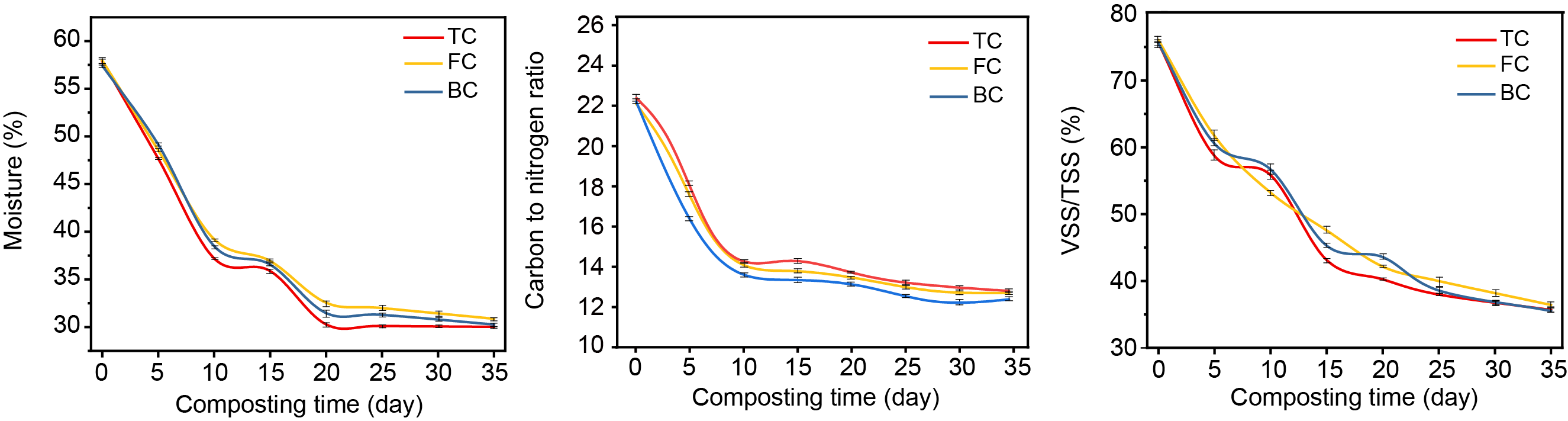


**Fig S3** Moisture, carbon to nitrogen ratio, and VSS/TSS of temporal composting samples.


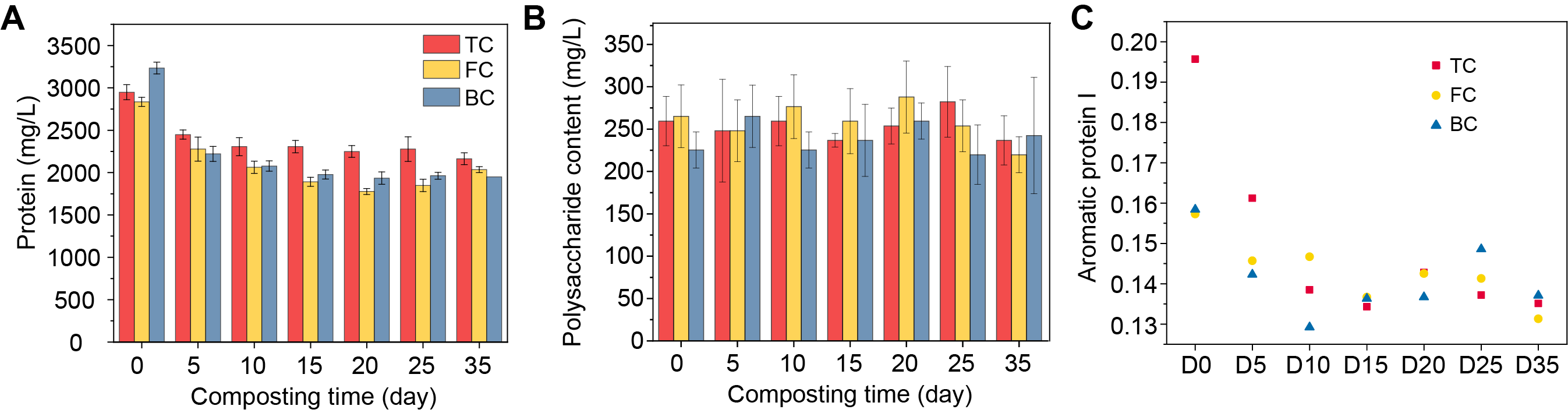


**Fig S4** (A) Protein and (B) polysaccharide contents in composting samples, (c) dynamics of aromatic protein I in composting samples.


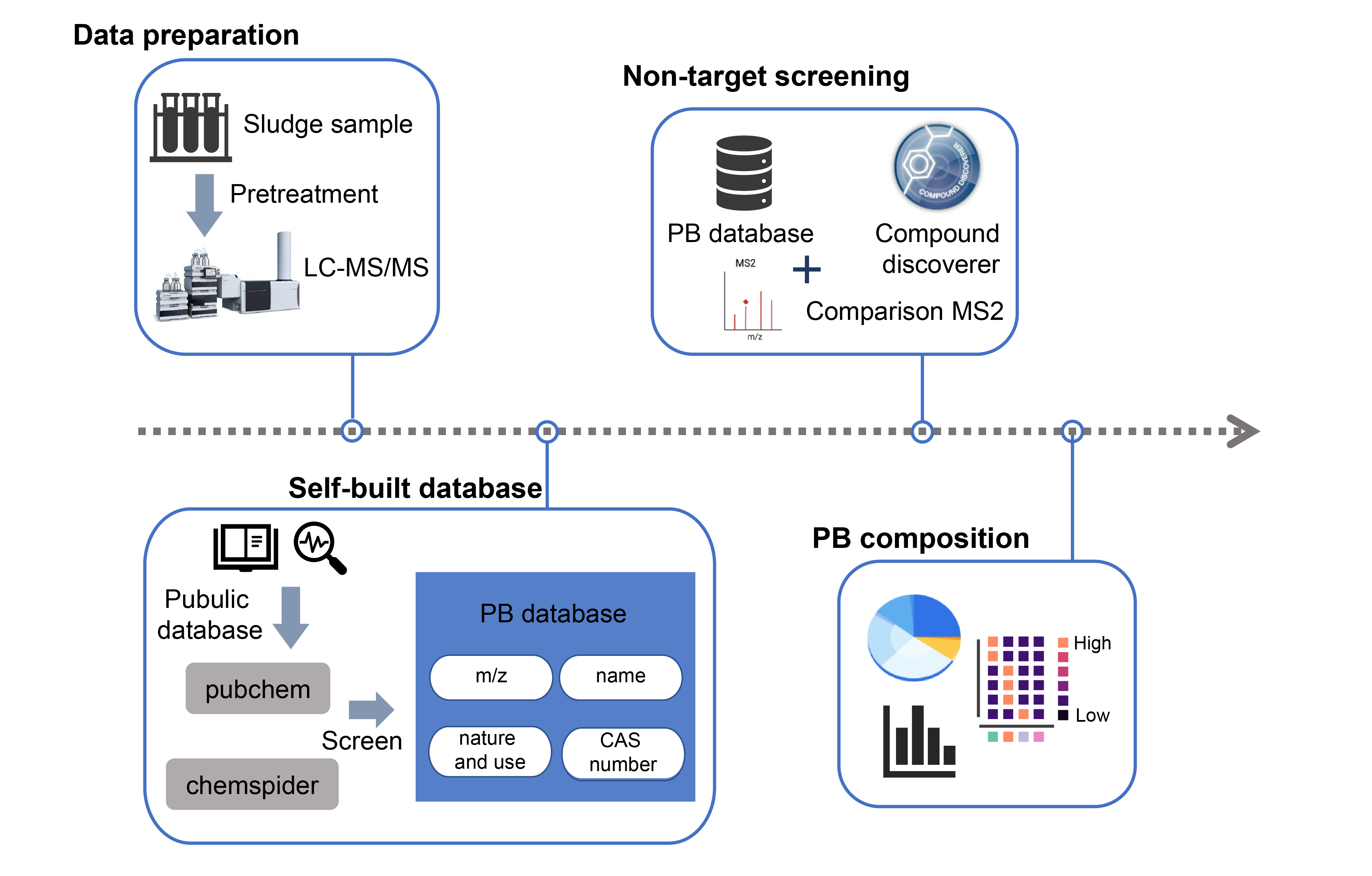


**Fig S5** Non-targeted identification of PB workflow.





**Fig S6** Upset diagram of PB composition and a list of certain PB molecules in different groups.





**Fig S7** Relative content and classification of PB. Red stars and red fonts indicate D-PBs


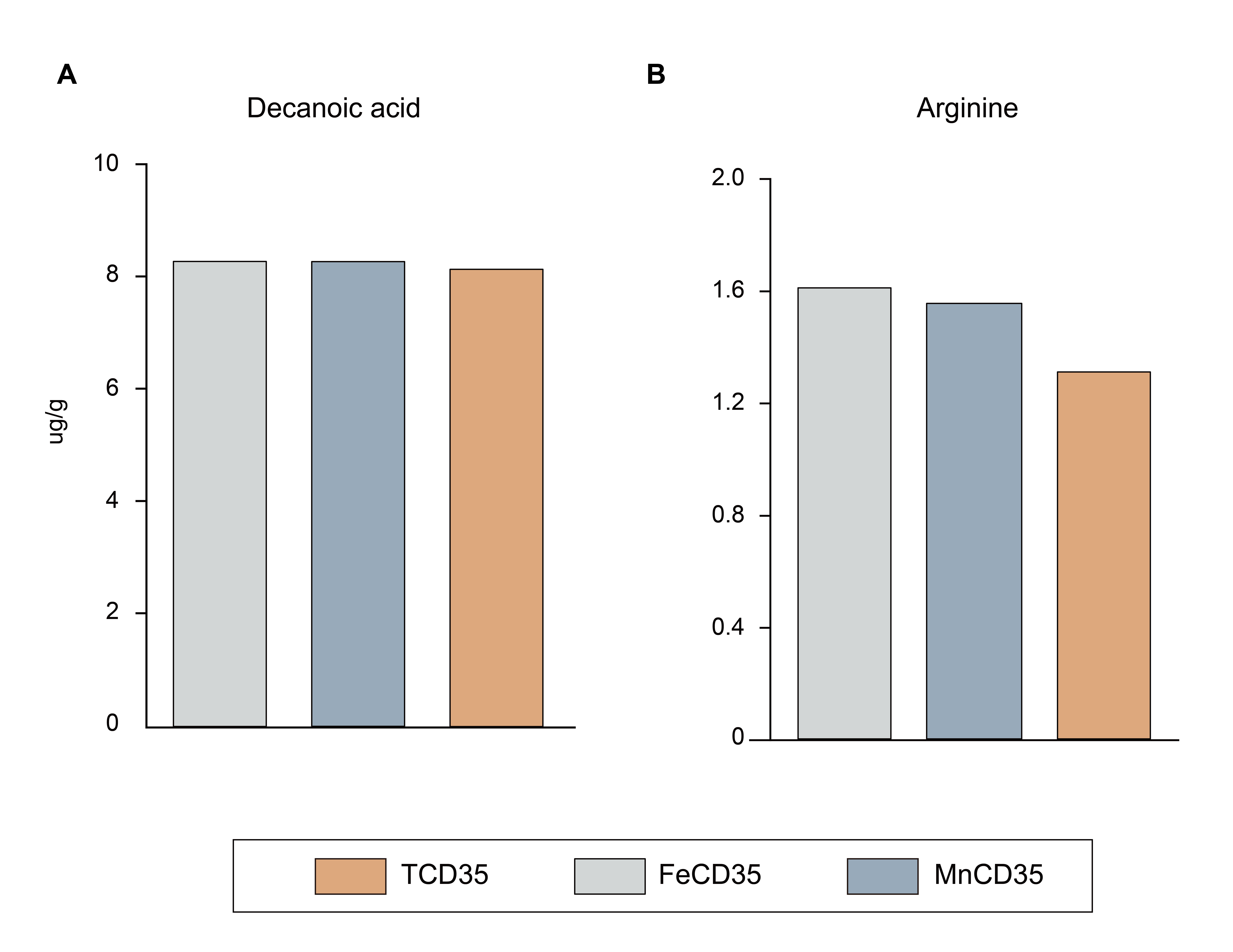


**Fig S8** Decanoic acid and Arginine content in three composting samples.


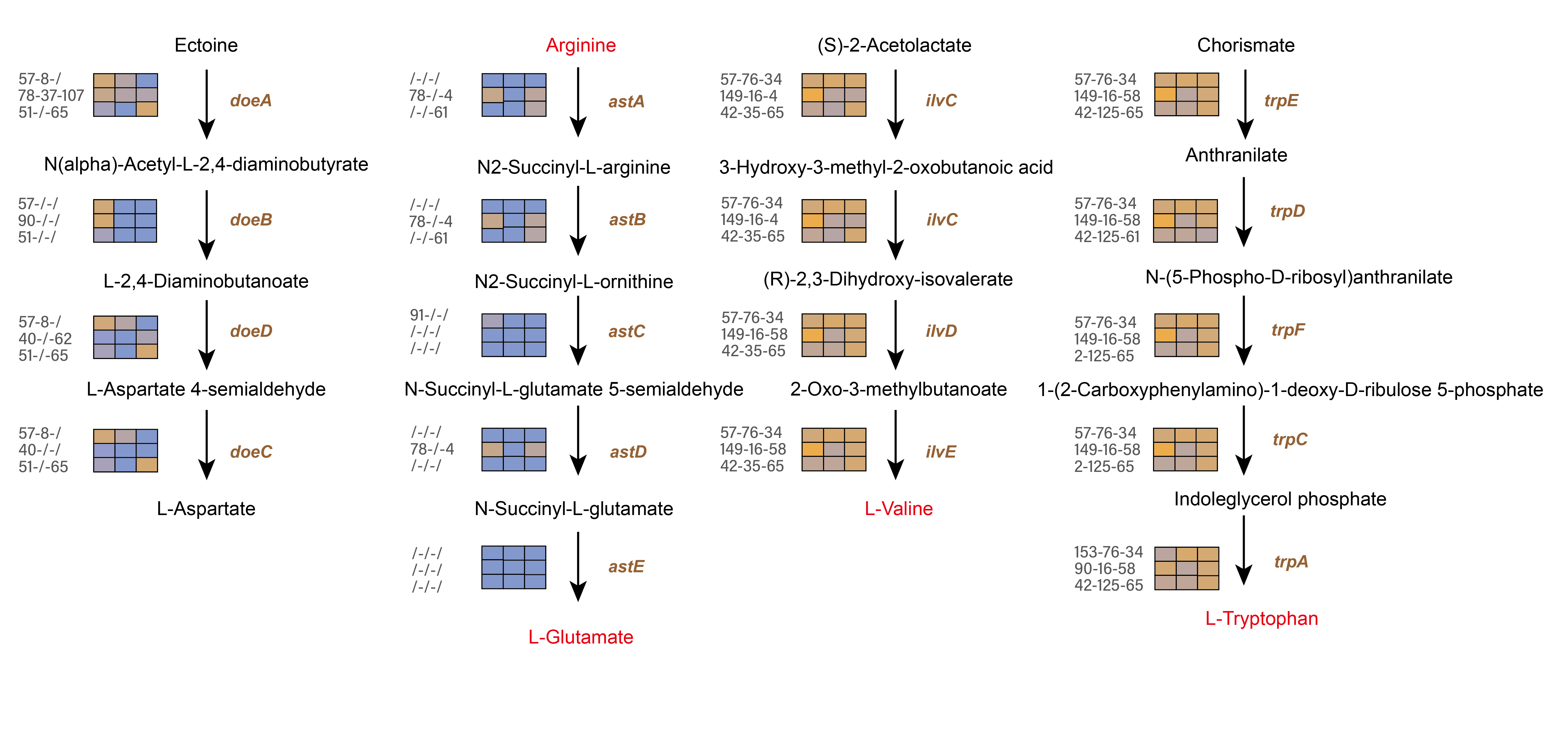
 **Fig S9** Indirect synthetic paths in D-PB.


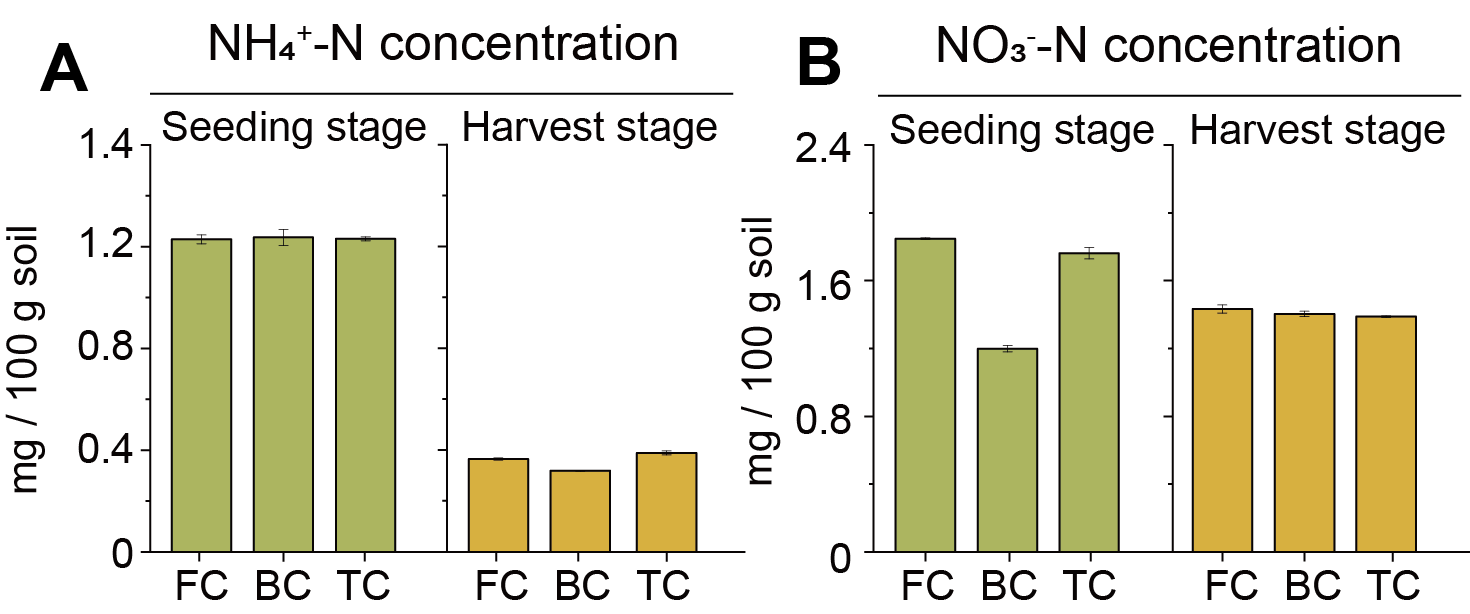


**Fig S10** (A) NH4+-N and (B) NO3—N concentrations of soils amended with TC, FC, and BC before and after pot experiments.


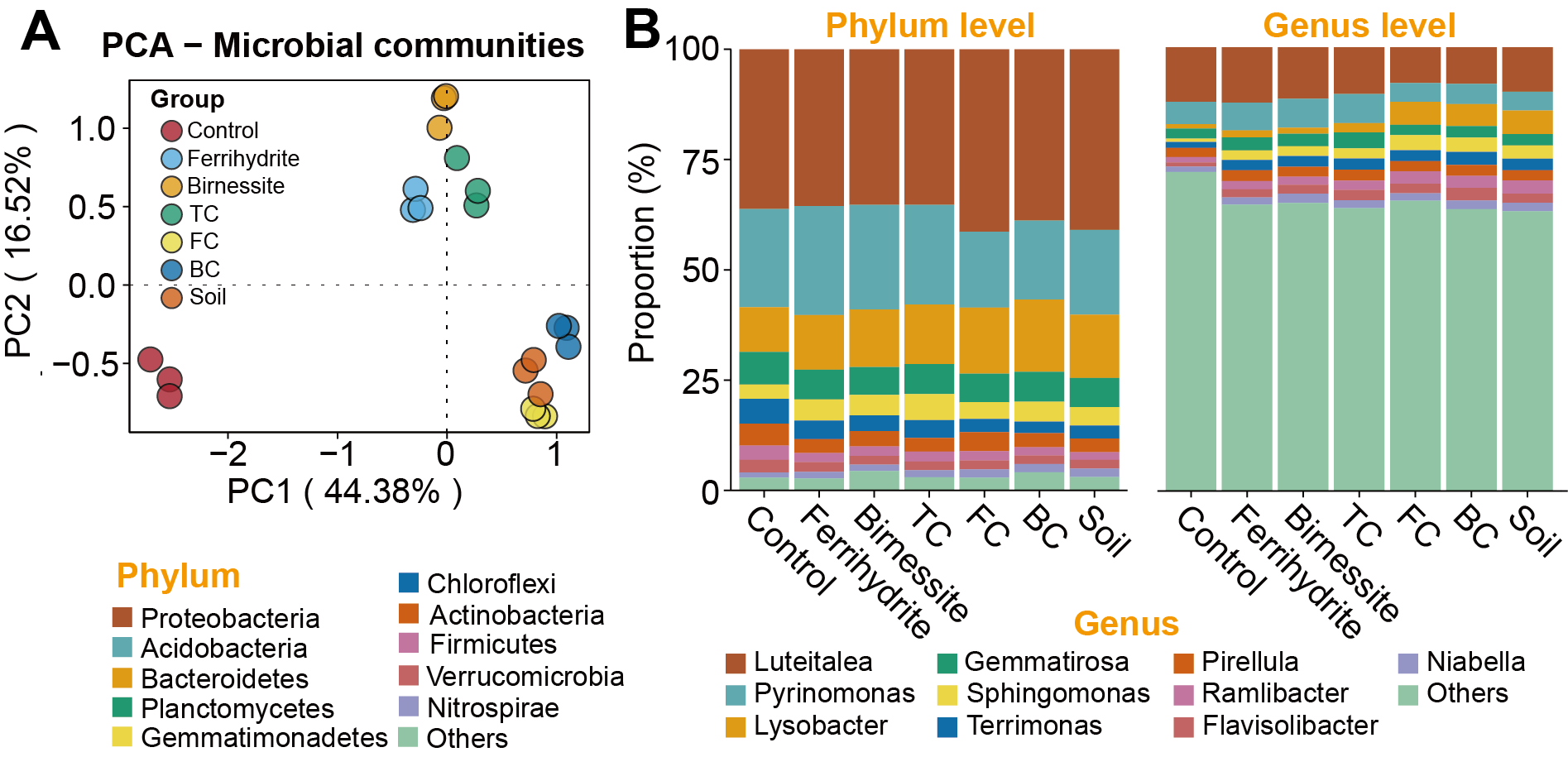


**Fig S11** (A) PCA analysis and (B) microbial communities of rhizosphere microorganisms in pots at harvest stage.

**Table S1** The location information of used feedstock in this study

| **Feedstock** | **City** | **longitude** | **Latitude** |
| --- | --- | --- | --- |
| RAS | Nanjing, China | 118°46'43''E | 32°02'38''N |
| Corn straw | Jiangsu, China | 118°34′5.5″E | 34°28′44″N |

**Table S2** Basic parameters of used feedstock in this study

| **Feedstock** | **Moisture (%)** | **Organic matter (%)** | **pH** | **Ec (μS/cm)** |
| --- | --- | --- | --- | --- |
| Sludge | 82.44 | 60.51 | 6.96 | 508 |
| Corn straw | 10.77 | 85.37 | 6.41 | 2160 |

**Table S3** Relative content

| TC | FC/TC or BC/TC | D-PB |
| --- | --- | --- |
| 0 | >0.01 | IAA |
| ≠0 | >1.3 | Arginine, L-Pyroglutamic acid, Indole, Decanoic acid, trans-Aconitic acid |
|  | nearly disappears | Valine, Tryptophan |

**Table S4** p-values of differentially abundant genes

| gene | p-values | gene | p-values |
| --- | --- | --- | --- |
| *argG* | 0.027 | *gdh* | 0.044 |
| *argH* | 0.022 | *fadB* | 0.031 |
| *doeA* | 0.035 | *fabG* | 0.033 |
| *doeB* | 0.014 | *fabA/Z* | 0.034 |
| *doeC* | 0.032 | *fabI/K/V* | 0.029 |
| *doeD* | 0.033 | *fabB/F* | 0.028 |
| *astA* | 0.033 | *fabD* | 0.021 |
| *astB* | 0.034 | *ACCs* | 0.045 |
| *astC* | 0.025 | *MCH* | 0.047 |
| *gluC* | 0.017 | *tnaA* | 0.039 |
| *ilvB* | 0.016 | *ALDH* | 0.038 |
| *ilvC* | 0.016 | *DDC* | 0.036 |
| *ilvD* | 0.015 |  |  |
| *ilvE* | 0.019 |  |  |
| *trpE* | 0.028 |  |  |
| *trpA* | 0.024 |  |  |
| *trpC* | 0.025 |  |  |
| *trpD* | 0.027 |  |  |
| *trpF* | 0.027 |  |  |
| *adi1* | 0.047 |  |  |

**Text S1.** Detailed Methods for Sample Processing, Sequencing, and Bioinformatics Analysis

In brief, Raw sequence reads underwent quality trimming using Trimmomatic v0.36 (<http://www.usadellab.org/cms/uploads/supplementary/Trimmomatic>) to remove adaptor contaminants and low-quality reads. Qualified reads were assembled using MEGAHIT (v1.2.9), and the allowed minimal length for contigs was 1000 bp. Binning was then performed using MaxBin2 (v2.0), Metabat2 (v2.12.1), and CONCOCT (v1.1.0), followed by bin refinement by MetaWRAP (v1.3.0).
